# Exploration of Client Experiences of Veterinary Care for Companion Animals (Dogs and Cats) and the Impact of Trauma-Informed Care on Client Outcomes

**DOI:** 10.3390/vetsci12080709

**Published:** 2025-07-28

**Authors:** Vanessa I. Rohlf, Nadia Manfrenuzzi, Neelofar Rehman, Pauleen C. Bennett

**Affiliations:** School of Psychology and Public Health, La Trobe University, Bendigo, VIC 3552, Australiapauleen.bennett@latrobe.edu.au (P.C.B.)

**Keywords:** veterinary–client relationship, client communication, holistic care, veterinary practice, client education, client–provider relationship, patient care

## Abstract

Trauma-informed care (TIC) is an increasingly implemented approach in human services, but little research documents implementation within veterinary services. This study explored emotional experiences of veterinary clients and the perceived emotions/behaviors of their pets, studied experiences of TIC, and ascertained the extent to which experiences of TIC predicted client outcomes. Clients (*N* = 274) were surveyed about their experiences of veterinary care. Clients documented positive and negative emotional experiences, with negative emotions associated with more serious presenting issues. TIC was experienced during veterinary visits. Principal component analyses found that TIC fell into the following five factors: (1) transparent and client-centered communication; (2) client supports and strengths; (3) emotional safety and empowerment; (4) physical safety and comfort; (5) informed consent. Hierarchical regressions revealed that, after controlling for reason for the visit, client-reported emotions and pet emotions/behaviors, 53.9% and 56.3% of the variance in client satisfaction and client disappointment was accounted for by TIC practices, respectively. The results show that although the outcomes for pets receiving veterinary care might not always be positive, which can lead to negative emotional experiences for clients, these can be ameliorated and lead to positive client outcomes through utilizing TIC.

## Simple Summary

Trauma-informed care (TIC) is an approach often utilized in human services, but little is known about its implementation in veterinary services. This study explored the emotional experiences of clients visiting veterinary clinics/hospitals, perceived emotions/behaviors of their pets, and experiences of TIC. Whether or not TIC predicted client outcomes was also explored. Results from an online anonymous survey of 274 participants revealed that positive and negative emotions were experienced during visits to the veterinary clinic/hospital, with negative emotions being linked with more serious presenting issues. TIC was experienced by clients during veterinary visits and was best characterized by five features: (1) transparent and client-centered communication; (2) client supports and strengths; (3) emotional safety and empowerment; (4) physical safety and comfort; (5) informed consent. Further analyses revealed that TIC significantly predicted client satisfaction with quality of care and client disappointment, even after controlling for the possible influence of reason for the visit, and clients’ reported emotions and pet emotions/behaviors. These results demonstrate that although outcomes for pets receiving veterinary care might not always be positive, which leads to negative emotional experiences for clients, these can be ameliorated and lead to positive client outcomes through implementing TIC.

## 1. Introduction

Trauma can be described as an occurrence or a series of events that an individual experiences as physically or emotionally damaging, and that has long-lasting negative effects on social, spiritual, mental, physical, and emotional wellbeing [1,2]. It can take many forms, including acute trauma, consisting of exposure to a single incident (e.g., an accident), chronic trauma, where exposure occurs over an extended period (e.g., bullying), or complex trauma, where exposure is varied and consists of multiple events that are often interpersonal in nature (e.g., childhood abuse) [3]. Trauma can also be characterized as primary, where one has experienced the event directly, or secondary, where it is experienced by witnessing the event, hearing accounts of the event, or caring for those who have experienced trauma [3]. Trauma not only impacts individuals but, in some circumstances, can affect whole communities and generations [3].

While quite a narrow definition exists for the purpose of formally diagnosing clinically significant responses to trauma, such as posttraumatic stress disorder, broader definitions acknowledge that many individuals without formal diagnoses experience distress and long-lasting consequences from experiencing trauma. For example, a survey administered to 68,894 individuals across 24 countries found that 70% of respondents reported exposure to at least one traumatic event in their lifetime [4]. Understanding common consequences of exposure to trauma is particularly relevant to the veterinary profession for several reasons.

First, the circumstances that lead clients to seek veterinary services may be traumatic. Events affecting animals, such as motor vehicle accidents, penetrating injuries, illnesses, and animal bites, are common reasons for presentation in small animal emergency clinics/hospitals [5]. Second, for many companion animals, the experience of receiving veterinary care and undergoing medical procedures can be traumatic [6,7]. Dogs, for example, often display behavior indicative of fear, stress, and anxiety [8]. Given the close bond clients often have with their companions, witnessing their perceived suffering can be highly distressing [9]. Third, even if an animal is accepting of veterinary care, there are psychological costs associated with caring for those who are unwell, injured, or geriatric with multiple comorbidities, including mobility restrictions [10,11,12,13]. This toll has been referred to as caregiver burden, and it has been documented in clients whose companion animals experience a range of chronic conditions, such as cancer [14], behavior problems [15], skin conditions, diabetes, and gastrointestinal disease [12]. Fourth, for some clients, a veterinary visit may be associated with the death of a companion animal, which can cause significant distress. In a study of 177 companion animal owners, 70% reported grief following the loss of their companion animal, and 30% reported severe levels of grief in anticipation of, or after, the death of their pet [16]. A portion of companion animal owners report post-traumatic stress symptoms following the death of their companion animal [17,18,19].

Individuals with a history of childhood trauma are at greater risk of experiencing distress following the death of their companion animal [20] and may also be at higher risk of re-traumatization during non-lethal veterinary consultations. This is important to acknowledge since, given the high prevalence of exposure to trauma in the general population, it is highly likely that trauma is also prevalent among those seeking veterinary services. In fact, the bond between an individual and their pet may be particularly strong for those who have previously experienced trauma, due to pets providing safe and secure attachments, as well as emotional and psychological support [21,22]. Stronger bonds may result in more visits to veterinary clinics or higher expectations regarding the level of care provided.

The impact of trauma varies depending on an individual’s coping skills, experience and current social supports, but trauma typically affects individuals emotionally, physically, cognitively, and behaviorally [2]. Individuals impacted by trauma may have difficulty managing emotions, making decisions, or they may withdraw or become argumentative [3]. Responses to trauma may, therefore, impact how a client engages with veterinary services. While these behaviors may represent a client’s best attempts to cope with what has happened, or is happening, to them [3], this may lead to clients being perceived as “difficult”, “problematic”, or “needy” by veterinary staff.

Interactions with clients in distress or exhibiting “difficult” behaviors can be stressful for veterinary professionals. Caring for clients and their pets place veterinary staff at risk of workplace stress, burnout, and secondary traumatic stress [23,24,25,26]. Veterinary professionals also report being verbally and physically assaulted by clients, which has led to self-reported feelings of depression, decreased levels of job enjoyment, and increased intentions to leave the profession [27]. Communications around delivering difficult news can be particularly challenging [28,29]. Despite this, a systematic literature review, conducted in 2020, found that communication education was relatively absent in veterinary curricula [30]. Although changes may have been made to curricula in some regions since the date of publication, this reported absence is unfortunate since communication difficulties with clients can lead to negative health outcomes for animals and be a source of complaints against veterinarians [30]. The past decade has seen the introduction of important strategies to reduce stress and risk of trauma in companion animals in veterinary clinic/hospital settings [8]. Considerably less focus has been directed toward client levels of distress, their experiences of care and communication in the veterinary context, and how this influences outcomes.

Trauma-informed care (TIC) is an approach gaining momentum in human health, education, and welfare settings. It is a method of service delivery that acknowledges the high likelihood that individuals who seek services and those who work within these services have experienced trauma. In response, the approach seeks to construct settings that promote healing and growth and reduce the risk of further harm through re-traumatization [31]. While there are several TIC definitions and value-based principles proposed to guide implementation [32,33,34], an influential framework was developed by the Substance Abuse and Mental Health Services Administration (SAMHSA) [3]. According to SAMHSA, TIC must be embedded into policies, procedures, and practices and is best guided by the following six principles [3]:

(1) Safety: Clients and staff are entitled to feeling physically and psychologically safe;
(2) Trustworthiness and transparency: Operations and decisions are transparent to build trust among clients and staff;
(3) Peer support: Peer support and mutual self-help are leveraged and lived experiences guide improvements in service delivery;
(4) Collaboration and mutuality: Healing occurs through meaningful and inclusive relationships formed between clients and staff;
(5) Empowerment, voice, and choice: Operations empower staff and clients. Client strengths are acknowledged and shared decision making is promoted;
(6) Cultural, historical, and gender issues: Cultural biases and stereotypes are surpassed to address and recognize historical trauma.

TIC implementation within the human services field has documented positive outcomes. TIC is linked with greater motivation to comply with treatment recommendations, better health outcomes, and higher levels of satisfaction with the provision of services [35,36,37]. TIC is also linked with improvements in staff morale, staff wellbeing, and greater retention rates [38,39].

Little research exploring TIC in animal health and welfare settings has been conducted. TIC has been promoted as a means of managing work-related stress in the animal protection and welfare sector [40] and promoted as a useful concept in veterinary practice, particularly as a means of treating anxious and fearful patients [41]. How TIC is experienced and to what extent it is experienced by clients, as well as its subsequent impacts on client and patient outcomes, has not been described. Given the links between TIC and positive client outcomes within the human services sector, it might be assumed that similar benefits could be seen in the veterinary context. The aims in this study were to document the emotional experiences of veterinary clients and the perceived emotions/behaviors of their pets, explore veterinary clients’ experiences of TIC, and ascertain the extent to which the experience of TIC predicts client outcomes.

## 2. Materials and Methods

### 2.1. Study Design

A survey-based cross-sectional study design was used to explore client experiences of veterinary visits. The survey was part of a larger study exploring the experiences of veterinary care, which was approved by the La Trobe University human ethics committee (HEC24227). Participants were recruited via Prolific [42], an online data collection platform that researchers can access to connect with a diverse pool of participants who voluntarily agree to participate in research in exchange for a small fee. No identifying information was collected.

### 2.2. Participants

To be eligible to partake in this study, participants were required to be over 18 years old and understand English, the language of the survey. They were also required to have owned a pet and visited a veterinary clinic/hospital with their pet in the last 12 months. A total of 289 individuals commenced the survey, with 15 being eliminated due to non-completion, leaving a total sample of 274. Participant ages ranged from 18 to 65 years (*M* = 33.42 *SD* = 10.23). Participants identifying as a man or woman were relatively evenly represented in the sample, comprising 52.6% and 45.1% of the sample, respectively, but few individuals identified as third gender or non-binary (1.5%) and (0.8%) selected “rather not say”. As shown in Table 1, 29.1% resided in Australia, with the remaining participants residing in 27 other countries, most commonly South Africa, United States of America, and United Kingdom. Countries where less than 7 participants resided were included in an “Other” category. Most participants had a university education and reported their total household income, compared with others, as average or above average. Most pets were desexed male or female dogs, over 1 year of age. Just under half of veterinary visits were for preventative healthcare reasons and 72.6% of participants reported that the outcome of the visit was that their pet’s health was not affected or that they were fully recovered. However, 28.9% reported that they visited the veterinary clinic for a serious medical concern, and 8.4% reported that their pet had subsequently passed away.

### 2.3. Materials

The client and pet demographics reported in Table 1 were collected using a short questionnaire developed by the research team. Participants also completed items exploring their experiences relating to their most recent veterinary visit, including the reason for their visit, how recently the visit occurred, and the overall outcome. They were then asked to indicate the extent to which they had experienced a range of feelings during their last visit (5-point scales, where 1 = not at all and 5 = a great deal) and, if their pet had not been unconscious or under emergency care during the visit, the extent to which they perceived that their pet experienced a range of similar emotions.

Experiences of TIC were explored using a modified version of the Trauma-Informed Care—Patient Survey [43] with permission from the author. This 30-item scale asked participants to rate their level of agreement or disagreement (5-point scales, where 1 = strongly disagree and 5 = strongly agree) with a set of statements measuring features of care congruent with SAMHSA’s [3] core principles. This included client perceptions of emotional and physical safety, as well as collaboration and cultural sensitivity.

Client outcomes were measured by nine items developed by the research team. Participants were asked to indicate their level of agreement or disagreement with a set of statements exploring their overall level of satisfaction or disappointment with the veterinary care received (5-point scales, where 1 = strongly disagree and 5 = strongly agree).

### 2.4. Procedure

The questionnaire took approximately 20 min to complete. Participants were first asked to read a participant information statement and provide consent before commencing the survey. Data collection occurred during the month of August 2024, during several different time periods to ensure a variety of participants were sourced. After the first round of data collection, which was open to all Prolific users who met the selection criteria, it was found that only a small proportion of participants (7 of 211 or 3.32%) were from Australia, the authors’ country of residence. To ensure a greater representation of Australian residents, so the results could be relevant to local veterinary clinics, a second round of recruitment occurred, specifically targeting Australian Prolific users. This occurred over two days and resulted in an additional 79 participants. All participants were paid for their time in accordance with Prolific guidelines.

Analysis was conducted using SPSS version 29.0.2.0 [44]. Mean scores and standard deviations are reported for continuous variables, along with Cronbach’s alphas for subscales. Frequencies and percentages are reported for nominal and ordinal variables. To reduce the number of variables, summary variables were created for client emotional experiences, perceived pet emotions/behaviors, experiences of TIC, and client outcomes, by conducting principal component analyses (PCA) and Oblimin rotation. The KMO measure of sampling adequacy ranged from 0.845 to 0.901, while Bartlett’s test of sphericity was consistently significant. The cut-off for factor loadings was set at 0.40. Composite scores were created for the factors identified by taking the mean item score. For all subscales, high scores were indicative of a greater degree of identifying with feelings, TIC experiences, and client outcomes.

Prior to running correlations, variables were checked for normality using histograms, normal Q-Q plots, and Shapiro–Wilks tests for normality. Significant Shapiro–Wilks tests were found for all variables, but, because Shapiro–Wilks is sensitive to minor departures from normality in large samples, a visual inspection of graphs and plots was also conducted [45]. This indicated that items relating to clients’ negative emotional experiences were positively skewed; therefore, Spearman’s rho nonparametric correlations were used to explore associations among client emotional experiences, perceived pet emotions/behaviors, and the reason for the vet visit. The reason for the visit was ranked by seriousness of the presenting issue by the research team (1 = preventative veterinary care such as vaccinations, wellness check, diet advice; 2 = minor medical conditions such as ear infection, itching, follow-up aftercare; 3 = serious medical issues such as pet acutely ill, injured, end of life care).

Hierarchical multiple regression analyses were used to determine the extent to which client outcomes were predicted by TIC experiences after controlling for reason for the visit in Step 1, followed by client emotional experiences and perceived pet emotions/behaviors in Step 2. Prior to the interpretation of these analyses, several assumptions were checked, including assessment of normality, linearity, and homoscedasticity of residuals. Mahalanobis distance was used to identify multivariate outliers. Three outliers were identified using this method, but they were retained because Cook’s distance values were less than 1, indicating that these cases did not have a large impact on the models [45].

## 3. Results

### 3.1. Client Emotional Experiences

As presented in Table 2, participants reported experiencing a range of emotions during their most recent veterinary clinic visit. PCA found three factors which accounted for 77.23% of the variance. Factor loadings and descriptive statistics for subscales are presented in Table 2. Factor 1, which we classified as worried, described negative affect related to internalized feelings of anxiety, worry, and sadness. Factor 2, which we termed relaxed, described feelings of calmness and happiness, related to positive affect. Factor 3, which we labeled as frustrated, described negative affect related to externalized feelings of anger, confusion, and frustration. Subscales derived from the factors demonstrated good internal reliability, as demonstrated by Cronbach’s alpha levels above the recommended cut off of 0.70 [46]. The obtained range on all three subscales was as wide as possible. Table 2 suggests that clients experienced feeling relaxed and worried to a greater degree than being frustrated, with the mean score on the third factor falling below 2, indicating the emotions in this factor were felt “a little”. Mean scores for the other two factors were between 2 and 3, indicating that most items were felt “more than a little” by many participants.

### 3.2. Perceived Pet Emotions

Participants reported that their pet appeared to experience or display an array of emotions/behaviors during the veterinary visit. As can be seen in Table 3, PCA found two factors, which accounted for 68.34% of the variance in these items. Factor 1 described negative emotions/behaviors, including agitation and nervousness, so we labeled it agitated. Factor 2 described positive emotions/behaviors, including happy and friendly, so we called it happy. Subscales derived from the factors demonstrated good internal reliability, with Cronbach’s alpha levels above the recommended cut off of 0.70 [46]. Participants used the full range of responses, with mean scores on both subscales falling between 2 and 3, indicating that the feeling/behavior was mostly believed to have been experienced or displayed “a little” or “more than a little”.

### 3.3. Associations Between Clients’ Emotional Experiences, Perceived Pet Emotions/Behaivors, and Reason for Vet Visit

Spearman’s rho was used to explore associations among clients’ emotional experiences, their pets’ perceived emotions/behaviors during the veterinary visit, and the reason for the visit, which was ranked by level of seriousness.

As can be seen in Table 4, client worry was strongly and positively correlated with client frustration and both of these measures were moderately to strongly correlated with client relaxation. Moreover, client worry and frustration were both positively correlated, and client relaxation negatively correlated, with pet agitation; the more a client perceived their pet to be agitated, the more the client was worried and frustrated and the less they were relaxed. Client-perceived pet happiness was moderately positively associated with client relaxation but not significantly associated with client worry or frustration. Significant correlations were also found between client emotional experiences, perceived pet emotions/behaviors, and reasons for the vet visit. The more serious the presenting medical issue, the more clients reported experiencing both worry and frustration, and the less they experienced happiness. Pet agitation was positively associated with the seriousness of the presenting problem, and pet happiness negatively associated with this same variable, yet both of these correlations were quite weak.

### 3.4. Experiences of Trauma-Informed Care and Client Outcomes

A PCA found that TIC experienced in the vet clinic/hospital setting could best be summarized by five factors, which accounted for 57.10% of the variance. As shown in Table 5, Factor 1 described client experiences suggestive of a lack of transparent client-centered communication by clinic staff. These items were reverse-scored prior to subscale creation, so that the subscale measured greater desirable communication styles. Factor 2 captured experiences of staff identifying clients’ strengths and sources of support. Factor 3 referred to experiences that enhanced clients’ senses of emotional safety and empowerment. Factor 4 referred to client experiences related to physical safety and comfort. Factor 5 referred to client experiences of informed consent. Cronbach’s alpha levels for all subscales derived from these factors were above the recommended cut off of 0.70 [46]. Mean subscale scores indicated TIC features associated with transparency and client-centered communication (Mean = 4.39), and those associated with physical safety and comfort (Mean = 4.21) were common. Features associated with identification of strengths and supports displayed a subsequentially lower mean (Mean = 2.96).

A final PCA showed that client outcomes could best be summarized by two factors, which accounted for 73.80% of the variance. As shown in Table 6, Factor 1 described clients’ satisfaction with the quality of veterinary care and Factor 2 described clients’ disappointment regarding how they and their pet were treated. Cronbach’s alpha levels for the subscales derived from these factors were above the recommended cut off of 0.70 [46]. Mean subscale scores indicated that, for the most part, clients were satisfied with the quality of care received and with their overall treatment.

### 3.5. Predicting Client Outcomes from Reason for Visit, Client Emotional Experiences, Perceived Pet Emotions/Behaviors, and Trauma-Informed Care Practices

Results of the hierarchical multiple regressions are presented in Table 7. For client satisfaction with quality of care, seriousness of the presenting problem, entered in Step 1, explained 1.6% of the variance *F*(1, 267) = 4.26, *p* = 0.040. The addition of client worry, frustration and relaxation, and pet agitation and happiness, in Step 2, explained an additional 7.6% of the variance *F*(6, 262) = 4.41 *p* = 0.0001. In the final model, entry of the five TIC subscales explained a further 53.9% of the variance, *F*(11, 257) = 39.95, *p* = 0.0001. In the final model, three of the five TIC variables made unique significant contributions. These were transparent and client-centered communication, emotional safety and empowerment, and informed consent.

For client disappointment, seriousness of the presenting problem, entered in Step 1, explained 2.4% of the variance, *F*(1, 267) = 6.57, *p* = 0.01. The addition of client and pet emotional experiences in Step 2 explained an additional 13.1% of the variance, *F*(6, 262) = 8.14, *p* = 0.0001. In the final model, entry of the TIC subscales explained a further 56.3% of the variance, *F*(11, 257) = 59.40, *p* = 0.0001. Two variables made independent contributions to the model. These were one of the client emotion variables (client frustration) and one of the TIC variables (transparent and client-centered communication).

## 4. Discussion

There were two aims to this study. The first was to document the emotional experiences of clients, and the perceived emotions/behaviors of their pets, when visiting veterinary clinics/hospitals. The second was to explore client experiences of trauma-informed care and the extent to which these experiences predict client outcomes.

### 4.1. Client Emotional Experiences and Perceived Pet Emotions/Behaviors

Participants reported experiencing a range of emotions during their most recent veterinary visit. These could best be conceptualized as three factors, which we labeled relaxed, including feeling calm and happy; worried, referring to feelings of worry, powerlessness, sadness, and anxiety; and frustrated, which referred to feelings of confusion, anger, and frustration. Participants also reported that their pets exhibited a range of emotions/behaviors during their last visit to the vet. Positive pet emotions/behaviors, labeled as happy, referred to the extent to which pets were perceived as being happy, friendly, and calm, whereas negative pet emotions/behaviors, labeled as agitated, referred to the extent to which pets were perceived as being agitated, nervous, and fearful.

Overall, participants reported experiencing more positive than negative affect during their most recent visit, although scores on each subscale ranged from 1 to 5, indicating that at least some participants recalled a very negative experience. This may, in part, be due to the reason for the visit, with clients who reported visiting the vet for more serious issues reporting more negative affect than those presenting for less serious issues. This is consistent with previous findings. Shaw et al. [47] evaluated veterinarian–client–patient communication patterns during videotaped clinical interviews of problem and wellness appointments. They found that clients were rated as more anxious and distressed during problem appointments compared with wellness appointments. Clients were also rated as less warm and friendly and less respectful during problem appointments compared with wellness appointments. This confirms that attending veterinary clinics can be upsetting for some clients, particularly when the presenting issue is serious. High levels of negative affect may have negative implications for establishing rapport [47], particularly if these emotions are not ameliorated by sensitive and compassionate communication by the veterinary team. Negative affect may also impact the degree to which medical information is understood and retained [48,49,50], because clients may divert resources to managing stress whilst also processing complex information, which may lead to attention difficulties [48].

Demands on clients may be further exacerbated by the presence of negative pet behaviors such as agitation, whereby the client’s attention may be on managing their pet and their own emotions, rather than on processing complex medical information. Indeed, participants reported more negative than positive pet emotions/behaviors during their visit to the vet clinic. This corresponds with past research that suggests visits to the veterinary clinic are stressful for pets, particularly dogs and cats [8,51,52]. Consistent with expectations, negative pet emotions/behaviors were associated with negative client affect. This may, in part, be due to emotion contagion between the owner and their pet [53,54]; however, both clients and pets were understandably affected by the seriousness of the presenting problem, with more serious medical issues being associated with more negative owner emotions, particularly worry, and more agitation on the part of the pet.

While the nature of medical issues cannot be controlled, current efforts to minimize stress in pets attending clinics [8] may also have a positive impact on client emotions. Likewise, direct efforts to minimize negative emotions in clients may also reduce pet agitation. Veterinary visits are complex, and the overall experience of the visit is likely a combination of many factors, including the presenting issue and client interactions with the pet, other clients and, most importantly, veterinary team members [8,55]. Other factors, such as the physical environment and past experiences of clinic visits, also likely contribute to the veterinary visit experience, which may then impact client and patient outcomes [8,41]. For this reason, implementation of TIC, as is practiced in human service delivery contexts, may be of critical importance in minimizing negative affect and stress in clients, which could lead to an enhanced quality of care for patients [56].

### 4.2. Experiences of TIC and Client Outcomes

This study found that several components of TIC were commonly experienced during veterinary visits. TIC may, therefore, already be part of the care provided to clients and their pets in veterinary settings despite a lack of empirical research documenting its delivery within the sector. Variability in the perceived level of TIC provided to clients did exist, however, indicating that some clients experience TIC to a greater degree than others.

According to SAMHSA, there are six TIC principles: empowerment; peer support; safety; trustworthiness and transparency; cultural, historical, and gender issues; and collaboration and mutuality. These principles were originally designed to provide a framework for behavioral health specialty sectors, and they were derived through a thorough and comprehensive consultation process involving those with lived experience, policy makers, and researchers. While this means that they provide a suitable starting point to begin to characterize what TIC may look like in veterinary settings, our results suggest that experiences of TIC may look different in veterinary care settings compared with settings with a focus on humans only. We identified five rather than six factors, which we characterized as reflecting the following: transparent and client-centered communication; supports and strengths; emotional safety and empowerment; physical safety and comfort; and informed consent. Cultural, historical, and gender issues did not fall into a separate factor, with relevant items incorporated into transparent and client-centered care as well as supports and strengths.

The extent to which TIC was experienced by veterinary clients predicted client satisfaction with the quality of care received and dissatisfaction with overall treatment. After controlling for the impact of the presenting issue, client emotional experiences and perceived pet emotions/behaviors explained 53.9% and 56.3% of the variance, respectively. Client satisfaction with the quality of veterinary care was best predicted by three of the five TIC factors: transparent and client-centered communication, emotional safety and empowerment, and informed consent. This suggests that veterinary team members can play a role in enhancing satisfaction with the level of care provided by ensuring that their communications, policies, and procedures align with these key areas, as follows.

First, transparent and client-centered communication requires open communication with clients, including a non-judgmental approach to listening to the client, appropriate responding to non-verbal communication, clearly outlining treatment options, and respecting client diversity. An additional feature of TIC is that the treatment of the client should not remind them of past negative experiences. Veterinary team members may be made aware of past trauma exposure through direct and sensitive questioning and/or through reading of client records. However, they are unlikely to be aware of all client exposure to trauma, so all communications should be sensitive to this possibility. This means that all interactions should align with trauma-informed principles regardless of whether trauma exposure has or has not been disclosed. Second, enhancing emotional safety and empowering clients may be achieved by equalizing power imbalances that exist between the client and veterinary team, taking responsibility for miscommunications, acknowledging that the client knows their situation and that of their pet best, and validating their experiences. Offering clients choices to fit their circumstances is also an important facet to enhancing emotional safety and empowerment for clients, which is consistent with a spectrum of care approach [57]. Third, informed consent can be achieved by emphasizing that any treatment is the client’s choice, checking their understanding of medical information, and seeking permission prior to performing medical activities. This can further empower clients when making considerably difficult decisions regarding their pet’s health. It must, however, also be acknowledged that there may be cases when it is inappropriate to treat animals in a certain way under some conditions, or to withhold treatment, and veterinary team members may refuse a request that does not align with their duty of care towards the patient, and where legislation prohibits certain approaches. These aspects of care can be discussed sensitively and openly with clients.

TIC experiences also predict client dissatisfaction with general treatment, which includes a lower likelihood of returning to the clinic. This shows that, when these features of care are not perceived to be provided by veterinary team members, they contribute to dissatisfaction, even after controlling for the impact of other variables. Ensuring that transparent client-centered communication is achieved is the most important TIC predictor of this outcome.

### 4.3. Limitations and Suggestions for Future Research

This research revealed potential relationships between client emotional experiences at the veterinary clinic/hospital, perceived pet emotions/behaviors, and seriousness of the presenting issue. It was also found that different levels of TIC are experienced by clients during their visit and that this predicts client satisfaction. A number of limitations should, however, be noted. The delivery of veterinary services around the world varies, and while this study does incorporate the perspectives of individuals from across the globe, there are some countries, like Australia, that are overrepresented in this sample, which limits the ability to generalize to all populations. This sample also has relatively high reported levels of education and income and, given that individuals who experience economic disadvantage are more likely to experience trauma and poorer outcomes following exposure to traumatic events [58], individuals with trauma exposure who are currently experiencing its impacts may be underrepresented in this sample. The impact of trauma-informed care practices on client outcomes may, therefore, be far greater than what is reported in this manuscript. Further to this, the size of the clinic and the resources and equipment available were not recorded or controlled for in this study. Future studies should explore the impact that clinic resourcing has on client satisfaction ratings. It should also be noted that this is a cross-sectional study based on client recall, and causality cannot be determined. Therefore, results may be subject to recall bias [59]. Future research documenting client experiences in real time, incorporating observations of pet behaviors and veterinary team member communications, are required to confirm and extend these findings.

TIC is a relatively new concept, with theoretical publications significantly outweighing empirical studies in the academic literature [60]. More research is required across all service delivery systems to clarify how TIC should best be operationalized and implemented. The importance of TIC is increasingly acknowledged in human services, due to a growing recognition of the pervasiveness of trauma and its impact on how clients engage with services. Given that veterinary care is a service utilized by these very same people, those who research communication in veterinary medicine and who practice in the field, as well as those with lived experiences, must be part of the future development of TIC. Indeed, the involvement of the veterinary profession may be critical, given that many of those affected by trauma are known to become reliant on pets for emotional support [21,22]. Veterinary staff awareness of trauma, as well as their views towards implementation of TIC in their practice, their levels of confidence, and their perceived barriers to practice all require additional research.

Research in the human services sector demonstrates that TIC is linked to positive outcomes for service providers as well as clients, leading to a reduction in burnout as well as decreased turnover [60]. Many veterinary professionals have their own personal histories of trauma [61]. Indeed, their past experiences may have contributed to their decision to enter a profession that involves animal care. Yet, through their work, they may be exposed to traumatic events. Secondary traumatic stress has been documented in veterinary nurses, technicians, and veterinarians, which is then linked with turnover intentions [24,25]. Future research is required to determine whether the implementation of TIC has a positive impact on staff mental health, wellbeing, and turnover.

## 5. Conclusions

Clients report experiencing a range of emotions when visiting veterinary services and they report more negative than positive pet emotions/behaviors. Trauma-informed care in the context of veterinary clinics/hospitals appears to include the provision of transparent and client-centered communication; a focus on identifying supports and client strengths; emotional safety and empowerment; physical safety and comfort; and informed consent. Some of these aspects of care, independent of the presenting issue, perceived pet emotions/behaviors, and client affect, contribute significantly to client outcomes, including satisfaction with the quality of veterinary care, the likelihood of returning to the clinic, and the client’s general perception of the veterinary setting. These findings demonstrate the importance of taking a whole veterinary clinic/hospital approach to care, including how clients and their pets are treated by all veterinary team members, and how veterinary service settings are built to accommodate the diverse needs of clients and their pets.

## Figures and Tables

**Table 1:** Frequencies and percentages of client, pet, and vet visit characteristics.

Variable			*n*	%
Client characteristics	Country of residence	Australia	80	29.2
		South Africa	43	15.7
		United States of America	28	10.2
		United Kingdom	27	9.9
		Poland	24	8.8
		Canada	15	5.5
		Other *	57	20.7
	Education	Primary	1	0.4
		Lower secondary	2	0.7
		Higher secondary	42	15.6
		Postsecondary, non-tertiary	35	13.0
		Undergraduate tertiary degree	135	50.2
		Postgraduate tertiary degree	54	20.1
	Household income	Much less than average	3	1.1
		Less than average	33	12.2
		Average	135	50.0
		Above average	73	27.0
		Much more than average	19	7.0
		Prefer not to say	7	2.6
Pet characteristics	Type of animal	Dog	182	67.4
		Cat	80	29.6
		Small mammal (rabbit, guinea pig, rat, mouse)	5	1.9
		Other (bird, fish, alpaca)	3	1.2
	Sex of animal	Female desexed	90	33.6
		Female not desexed	37	13.7
		Male desexed	90	33.3
		Male not desexed	53	19.6
	Age of pet	Under three months	8	3.0
		Three to six months	21	7.8
		Six to twelve months	35	13.0
		One to seven years	133	49.3
		Over seven years	73	27.0
Vet visit characteristics	Time of last visit	Within last week	14	5.1
		One to four weeks ago	58	21.2
		Four weeks to six months ago	127	46.4
		Six to twelve months ago	75	27.4
	Vet visit reason	Preventative healthcare	116	42.5
		Minor medical concern	78	28.6
		Serious medical concern	79	28.9
	Pet outcome	Health not affected	109	39.8
		Fully recovered	90	32.8
		Partially recovered	28	10.2
		Still receiving treatment	24	8.8
		Passed away	23	8.4

Note: * Other countries with *n* < 7 = Spain, Portugal, Chile, Greece, Hungary, Italy, Kenya, Mexico, Germany, India, Argentina, Belgium, Czech Republic, Finland, France, Croatia, Ireland, Japan, Republic of Korea, Netherlands, Sweden, United States Virgin Islands.

**Table 2:** Factor loadings based on principal component analysis (PCA) performed on client’s emotional experiences during the veterinary visit followed by descriptive statistics of subscales derived from the factors (*N* = 274).

Factor Loadings Following Rotation	Factor 1. Negative Affect— Worried	Factor 2. Positive Affect— Relaxed	Factor 3. Negative Affect— Frustrated
Worried for my pet	**0.956**	−0.010	0.101
Powerless	**0.783**	0.061	−0.182
Sad	**0.751**	−0.007	0.227
Anxious	**0.605**	−0.380	0.036
Calm	0.085	**0.941**	0.064
Happy	−0.119	**0.768**	−0.005
Confused	0.047	−0.017	**−0.873**
Angry	−0.050	−0.007	**−0.873**
Frustrated	−0.086	−0.037	**−0.792**
**Descriptive Statistics** **for Subscales**			
Median	2.25	2.50	1.33
Mean	2.56	2.74	1.70
Standard deviation	1.18	1.12	0.95
Possible range	1–5	1–5	1–5
Actual range	1–5	1–5	1–5
Cronbach’s alpha	0.889	0.725	0.843

**Table 3:** Factor loadings based on principal component analysis (PCA) performed on client-perceived pet emotions/behaviors during the veterinary visit followed by descriptive statistics of subscales derived from the factors (*N* = 271).

Factor Loadings Following Rotation	Factor 1. Negative Pet Emotion/Behavior—Agitated	Factor 2. Positive Pet Emotion/Behavior—Happy
Agitated	**0.817**	−0.015
Nervous	**0.813**	−0.150
Frightened	**0.776**	−0.178
Aggressive	**0.748**	0.205
Anxious	**0.680**	−0.166
Happy	0.068	**0.927**
Friendly	0.068	**0.828**
Calm	−0.014	**0.715**
**Descriptive Statistics for Subscales**		
Median	2.40	2.00
Mean	2.56	2.36
Standard deviation	0.98	1.00
Possible range	1–5	1–5
Actual Range	1–5	1–5
Cronbach’s alpha	0.855	0.816

**Table 4:** Spearman’s rho correlations between clients’ emotional experiences, pets’ perceived emotions/behaviors during the veterinary visit, and seriousness of the presenting issue.

	1	2	3	4	5	6
1. Pet happy	-					
2. Pet agitated	−0.553 **	-				
3. Client frustrated	−0.065	0.347 **	-			
4. Client worried	−0.255	0.457 **	0.687 **	-		
5. Client relaxed	0.495 **	−0.290 **	−0.458 **	−0.642 **	-	
6. Seriousness of presenting issue	−0.185 **	0.248 **	0.436 **	0.534 **	−0.451 **	-

Note: ** *p* < 0.01.

**Table 5:** Factor loadings based on principal component analysis (PCA) performed on clients’ trauma-informed care experiences during the veterinary visit followed by descriptive statistics of subscales derived from the factors (*N* = 274).

Factor Loadings Following Rotation	Factor 1. Transparent and Client Centered Communication	Factor 2. Client Supports and Strengths	Factor 3. Emotional Safety and Empowerment	Factor 4. Physical Safety and Comfort	Factor 5 Informed Consent
Staff responded inappropriately to my nonverbal communication (e.g., crying) ^b^	**0.853**	0.087	0.053	0.013	0.013
Staff treated me in a way that reminded me of a past negative experience where I felt disempowered or upset ^b^	**0.832**	0.042	0.002	0.049	−0.050
Staff did not listen to me with genuine interest and did not adequately pay attention to my concerns ^b^	**0.767**	0.048	0.029	−0.168	−0.060
Staff did not communicate openly and failed to provide all relevant information about my pet’s medical issues ^b^	**0.757**	−0.059	−0.091	0.009	0.061
Staff did not adequately involve me in decisions surrounding my pet’s care ^b^	**0.756**	−0.055	0.004	0.013	−0.113
Staff did not clearly outline what treatment option would require from me ^b^	**0.719**	−0.068	−0.130	−0.089	0.104
Staff appeared judgmental when I expressed my feelings or concerns ^b^	**0.655**	0.156	−0.111	−0.008	−0.133
Staff were not respectful of my unique circumstances (e.g., cultural identity, sexuality) ^b^	**0.606**	0.079	−0.135	−0.077	0.055
Staff spoke to me in a patronizing manner, using negative language and criticism ^b^	**0.579**	0.219	−0.033	−0.167	−0.169
Staff did not adequately help me identify my challenges and difficulties that may get in the way of treatment ^b^	**0.551**	−0.399	−0.020	0.005	0.036
Staff provided me with information about other service providers in my community that could help me and my pet (e.g., support groups, counseling services or social workers, pet rehabilitation services, pet care services, fear free groomers)	0.131	**0.732**	0.023	0.101	0.133
Staff asked me about my cultural beliefs in relation to care for my pet and respected any cultural preferences for treatment	0.270	**0.671**	0.151	0.027	0.014
Staff did not ask me about any negative/stressful experiences at a veterinary clinic in the past ^b^	0.113	**−0.667**	0.216	−0.117	0.037
Staff inquired about others who may be helpful to include in my pet’s care (e.g., family member)	−0.011	**0.649**	0.124	−0.166	0.147
Staff helped me identify my strengths and skills that may facilitate management of my pet’s care	0.045	**0.611**	0.320	0.041	0.089
Staff found out what was most pressing for me in a kind and caring manner	−0.123	−0.091	**0.784**	−0.140	−0.095
Staff recognized and equalized power imbalances by understanding that I was relying on them for help	0.086	0.108	**0.653**	0.124	0.047
Staff provided me with choices that fit my life circumstances for treatment preferences	−0.086	0.055	**0.606**	0.026	0.041
Staff empowered me by acknowledging that I know best about my pet and my situation	−0.109	0.164	**0.601**	0.021	0.050
Staff made me feel welcome by being warm and friendly (e.g., using a welcoming tone of voice)	−0.081	−0.111	**0.556**	0.388	0.085
Staff explained everything in a way that I could understand by using plain language without jargon	−0.236	−0.141	**0.487**	0.116	0.227
Staff made me feel emotionally safe by providing reassurances and validating my experiences	−0.065	0.031	**0.454**	0.205	0.194
Staff acknowledged and took responsibility for any miscommunication	0.008	0.216	**0.403**	−0.149	.277
Staff responded in a caring manner if I disclosed any past stressful experiences ^a^	−0.343	0.284	0.398	−0.062	−0.029
The physical space inside the clinic was comfortable for me and my pet (e.g., comfortable chairs, air conditioning)	0.132	0.057	0.355	**0.739**	0.007
The physical space inside the clinic felt unsafe for me and my pet (e.g., no ability to separate nervous or agitated pets from other animals) ^b^	0.308	−0.113	0.185	**−0.686**	−0.022
The physical space outside the clinic felt unsafe for me and my pet (e.g., inadequate parking, poor lighting, unsafe access) ^b^	0.404	−0.077	0.177	**−0.587**	0.074
Staff used statements (e.g., it is your decision, it is not for me to decide) that clearly explained my role in making decisions for my pet	0.061	−0.034	−0.151	−0.037	**0.830**
Staff checked for my understanding of information provided before proceeding	−0.101	0.101	0.168	0.135	**0.668**
Staff sought my permission prior to performing any medical activities (e.g., touching my pet or any medical examinations)	−0.194	0.217	0.128	−0.081	**0.486**
**Descriptive Statistics for Subscales**					
Median	4.50	3.00	3.67	4.33	3.67
Mean	4.39	2.96	3.83	4.21	3.65
Standard deviation	0.63	0.89	0.58	0.72	0.76
Possible range	1–5	1–5	1–5	1–5	1–5
Actual range	1–5	1–5	1.88–5	1.33–5	1–5
Cronbach’s alpha	0.909	0.775	0.826	0.729	0.593

Note: ^a^ Item not included in subscales. The cut-off for factor loadings set at 0.40. ^b^ Items reverse-scored before computing subscales.

**Table 6:** Factor loadings based on principal component analysis (PCA) performed on client-rated outcomes followed by descriptive statistics of subscales derived from the factors (*N* = 274).

Factor Loadings Following Rotation	Factor 1. Satisfaction with Quality of Veterinary Care	Factor 2. Dissatisfaction with General Treatment
Overall, I left the clinic feeling confident about the outcome(s) for my pet	**0.909**	0.178
Overall, the level of care provided for my pet by staff was excellent	**0.808**	−0.133
Overall, the level of care provided for me by staff was excellent	**0.766**	−0.143
Overall, the outcome(s) for my pet was as good as possible, given the circumstances	**0.750**	−0.030
Overall, I would recommend this veterinary practice to others	**0.748**	−0.116
Overall, I would not return to this veterinary practice for future pet-related issues.	0.026	**0.936**
Overall, I was disappointed with how my pet was treated	−0.002	**0.906**
Overall, I was disappointed with how I was treated	−0.009	**0.896**
Overall, I wish I had gone to a different veterinary clinic	−0.099	**0.773**
**Descriptive Statistics for Subscales**		
Median	4.20	1.00
Mean	4.21	1.49
Standard deviation	0.66	0.71
Possible range	1–5	1–5
Actual range	1.60–5	1–5
Cronbach’s alpha	0.879	0.913

**Table 7:** Beta coefficients from linear multiple regressions predicting client outcomes from trauma-informed care practices.

		Client Outcomes	
		Satisfaction	Disappointment
Reasons for visit	Reason (1 = Preventative, 2 = Minor, 3 = Serious)	0.025	0.032
Client emotional experiences	Worried	−0.093	0.039
	Relaxed	0.094	−0.001
	Frustrated	−0.091	0.116 *
Perceived pet emotion/behaviors	Agitated	0.118 *	−0.054
	Happy	0.031	−0.020
Trauma-informed care practice	Transparent and client centered communication	0.194 ***	−0.7050 ***
	Client supports and strengths	0.032	−0.050
	Emotional safety and empowerment	0.51 ***	−0.051
	Physical safety and comfort	0.089	−0.082
	Informed consent	0.142 **	−0.044

Note: * *p* < 0.05, ** *p* < 0.01, *** *p* < 0.001.

## Data Availability

Data are unavailable due to privacy restrictions.

## References

[1] Substance Abuse and Mental Health Services Administration (2023). Practical Guide for Implementing a Trauma-Informed Approach.

[2] Briere L.N., Scott C. (2015). Principles of Trauma Treatment: A Guide to Symptoms, Evaluation, and Treatment.

[3] Huang L.N., Flatow R., Biggs T., Afayee S., Smith K., Clark T., Blake M. (2014). SAMHSA’s Concept of Truama and Guidance for a Trauma-Informed Approach.

[4] Kessler R.C., Aguilar-Gaxiola S., Alonso J., Benjet C., Bromet E.J., Cardoso G., Degenhardt L., de Girolamo G., Dinolova R.V., Ferry F. (2017). Trauma and PTSD in the WHO world mental health surveys. Eur. J. Psychotraumatol..

[5] Valtolina C. Triage of the trauma patient. Proceedings of the World Small Animal Veterinary Association World Congress Proceedings.

[6] Tateo A., Zappaterra M., Covella A., Padalino B. (2021). Factors influencing stress and fear-related behaviour of cats during veterinary examinations. Ital. J. Anim. Sci..

[7] Riemer S., Heritier C., Windschnurer I., Pratsch L., Arhant C., Affenzeller N. (2021). A review on mitigating fear and aggression in dogs and cats in a veterinary setting. Animals.

[8] Edwards P.T., Smith B.P., McArthur M.L., Hazel S.J. (2019). Fearful Fido: Investigating dog experience in the veterinary context in an effort to reduce distress. Appl. Anim. Behav. Sci..

[9] Whipple E.E. (2021). The Human–animal bond and grief and loss: Implications for social work practice. Fam. Soc..

[10] Spitznagel M.B., Jacobson D.M., Cox M.D., Carlson M.D. (2017). Caregiver burden in owners of a sick companion animal: A cross-sectional observational study. Vet. Rec..

[11] Spitznagel M.B., Jacobson D.M., Cox M.D., Carlson M.D. (2018). Predicting caregiver burden in general veterinary clients: Contribution of companion animal clinical signs and problem behaviors. Vet. J..

[12] Spitznagel M.B., Gober M.W., Patrick K. (2023). Caregiver burden in cat owners: A cross-sectional observational study. J. Feline Med. Surg..

[13] Kogan L.R., Wallace J.E., Hellyer P.W., Carr E.C.J. (2022). Canine caregivers: Paradoxical challenges and rewards. Animals.

[14] Shaevitz M.H., Tullius J.A., Callahan R.T., Fulkerson C.M., Spitznagel M.B. (2020). Early caregiver burden in owners of pets with suspected cancer: Owner psychosocial outcomes, communication behavior, and treatment factors. J. Vet. Intern. Med..

[15] Buller K., Ballantyne K.C. (2020). Living with and loving a pet with behavioral problems: Pet owners’ experiences. J. Vet. Behav..

[16] Adams J. (2021). Pet Death as Disenfranchised Loss: Examining Posttraumatic Growth and Attachment in College Students. Ph.D. Thesis.

[17] Adrian J.A.L., Deliramich A.N., Frueh B.C. (2009). Complicated grief and posttraumatic stress disorder in humans’ response to the death of pets/animals. Bull. Menn. Clin..

[18] Adrian J.A.L., Stitt A. (2017). Pet loss, complicated grief, and post-traumatic stress disorder in Hawaii. Anthrozoös.

[19] Brown O.K., Symons D.K. (2016). “My pet has passed”: Relations of adult attachment styles and current feelings of grief and trauma after the event. Death Stud..

[20] Ahn J., Lee S.W., Kim K., Jin B., Chung U.S. (2023). The relationship between childhood trauma experience and complicated grief: The importance of psychological support for individuals coping with pet loss in Korea. J. Korean Med. Sci..

[21] Bergen-Cico D., Smith Y., Wolford K., Gooley C., Hannon K., Woodruff R., Spicer M., Gump B. (2018). Dog ownership and training reduces post-traumatic stress symptoms and increases self-compassion among veterans: Results of a longitudinal control study. J. Altern. Complement. Med..

[22] Lass-Hennemann J., Schäfer S.K., Sopp M.R., Michael T. (2022). The relationship between attachment to pets and mental health: The shared link via attachment to humans. BMC Psychiatry.

[23] Pohl R., Botscharow J., Böckelmann I., Thielmann B. (2022). Stress and strain among veterinarians: A scoping review. Ir. Vet. J..

[24] Rohlf V.I., Scotney R., Monaghan H., Bennett P. (2022). Predictors of professional quality of life in veterinary professionals. J. Vet. Med. Educ..

[25] Chapman A.J., Rohlf V.I., Moser A.Y., Bennett P.C. (2024). Organizational factors affecting burnout in veterinary nurses: A systematic review. Anthrozoös.

[26] Wojtacka J., Grudzień W., Wysok B., Szarek J. (2020). Causes of stress and conflict in the veterinary professional workplace—A perspective from Poland. Ir. Vet. J..

[27] Rogers C.W., Murphy L.A., Murphy R.A., Malouf K.A., Natsume R.E., Ward B.D., Tansey C., Nakamura R.K. (2022). An analysis of client complaints and their effects on veterinary support staff. Vet. Med. Sci..

[28] Ballantyne K.C., Buller K. (2015). Experiences of veterinarians in clinical behavior practice: A mixed-methods study. J. Vet. Behav..

[29] Platt B., Hawton K., Simkin S., Mellanby R.J. (2012). Suicidal behaviour and psychosocial problems in veterinary surgeons: A systematic review. Soc. Psychiatry Psychiatr. Epidemiol..

[30] Pun J.K. (2020). An integrated review of the role of communication in veterinary clinical practice. BMC Vet. Res..

[31] Mahon D. (2022). Implementing trauma informed care in human services: An ecological scoping review. Behav. Sci..

[32] Brown T., Ashworth H., Bass M., Rittenberg E., Levy-Carrick N., Grossman S., Lewis-O’Connor A., Stoklosa H. (2022). Trauma-informed care interventions in emergency medicine: A systematic review. West. J. Emerg. Med..

[33] Harris M.E., Fallot R.D. (2001). Using Trauma Theory to Design Service Systems.

[34] Hopper E.K., Bassuk E.L., Olivet J. (2010). Shelter from the storm: Trauma-informed care in homelessness services settings. Open Health Serv. Policy J..

[35] Scheer J.R., Poteat V.P. (2021). Trauma-informed care and health among LGBTQ intimate partner violence survivors. J. Interpers. Violence.

[36] Hales T.W., Green S.A., Bissonette S., Warden A., Diebold J., Koury S.P., Nochajski T.H. (2019). Trauma-informed care outcome study. Res. Soc. Work. Pract..

[37] Ashby B.D., Ehmer A.C., Scott S.M. (2019). Trauma-informed care in a patient-centered medical home for adolescent mothers and their children. Psychol. Serv..

[38] Treisman K. (2024). Trauma-Informed Health Care: A Reflective Guide for Improving Care and Services.

[39] Hales T.W., Nochajski T.H., Green S.A., Hitzel H.K., Woike-Ganga E. (2017). An association between implementing trauma-informed care and staff satisfaction. Adv. Soc. Work..

[40] Stevenson R., Morales C. (2022). Trauma in animal protection and welfare work: The potential of trauma-informed practice. Animals.

[41] Corridan C.L., Dawson S.E., Mullan S. (2024). Potential benefits of a ‘trauma-informed care’ approach to improve the assessment and management of dogs presented with anxiety disorders. Animals.

[42] Prolific. www.prolific.com.

[43] Kokokyi S., Klest B., Anstey H. (2021). A patient-oriented research approach to assessing patients’ and primary care physicians’ opinions on trauma-informed care. PLoS ONE.

[44] IBM Corp (2023). IBM SPSS Statistics for Windows 29.0.2.0.

[45] Tabachnick B.G., Fidell L.S. (2007). Using Multivariate Statistics.

[46] DeVellis R.F., Thorpe C.T. (2021). Scale Development: Theory and Applications.

[47] Shaw J.R., Adams C.L., Bonnett B.N., Larson S., Roter D.L. (2008). Veterinarian-client-patient communication during wellness appointments versus appointments related to a health problem in companion animal practice. J. Am. Vet. Med. Assoc..

[48] Schwabe L., Wolf O.T. (2010). Learning under stress impairs memory formation. Neurobiol. Learn. Mem..

[49] Burke A., Heuer F., Reisberg D. (1992). Remembering emotional events. Mem. Cogn..

[50] Sharot T., Delgado M.R., Phelps E.A. (2004). How emotion enhances the feeling of remembering. Nat. Neurosci..

[51] Nibblett B.M., Ketzis J.K., Grigg E.K. (2015). Comparison of stress exhibited by cats examined in a clinic versus a home setting. Appl. Anim. Behav. Sci..

[52] Mariti C., Raspanti E., Zilocchi M., Carlone B., Gazzano A. (2015). The assessment of dog welfare in the waiting room of a veterinary clinic. Anim. Welf..

[53] Huber A., Barber A.L., Faragó T., Müller C.A., Huber L. (2017). Investigating emotional contagion in dogs (Canis familiaris) to emotional sounds of humans and conspecifics. Anim. Cogn..

[54] Yong M.H., Ruffman T. (2014). Emotional contagion: Dogs and humans show a similar physiological response to human infant crying. Behav. Process..

[55] Dawson L., Dewey C., Stone E., Guerin M., Niel L. (2016). A survey of animal welfare experts and practicing veterinarians to identify and explore key factors thought to influence canine and feline welfare in relation to veterinary care. Anim. Welf..

[56] Marsac M.L., Kassam-Adams N., Hildenbrand A.K., Nicholls E., Winston F.K., Leff S.S., Fein J. (2016). Implementing a trauma-informed approach in pediatric health care networks. JAMA Pediatr..

[57] Dolan E.D., Slater M.R. (2024). Veterinarians’ self-reported behaviors and attitudes toward spectrum of care practices. Animals.

[58] Abedzadeh-Kalahroudi M., Razi E., Sehat M. (2018). The relationship between socioeconomic status and trauma outcomes. J. Public Health.

[59] Colombo D., Suso-Ribera C., Fernández-Álvarez J., Cipresso P., Garcia-Palacios A., Riva G., Botella C. (2020). Affect recall bias: Being resilient by distorting reality. Cogn. Ther. Res..

[60] Bargeman M., Abelson J., Mulvale G., Niec A., Theuer A., Moll S. (2022). Understanding the conceptualization and operationalization of trauma-informed care within and across systems: A critical interpretive synthesis. Milbank Q..

[61] Strand E.B., Brandt J., Rogers K., Fonken L., Chun R., Conlon P., Lord L. (2017). Adverse childhood experiences among veterinary medical students: A multi-site study. J. Vet. Med. Educ..

